# CRISPR/Cas-Mediated Knockdown of PD-L1 and KRAS in Lung Cancer Cells

**DOI:** 10.3390/ijms25169086

**Published:** 2024-08-22

**Authors:** Summer A. Abounar, Nefertiti A. El-Nikhely, Kati Turkowski, Rajkumar Savai, Hesham Saeed

**Affiliations:** 1 Department of Biotechnology, Institute of Graduate Studies and Research, Alexandria University, Alexandria 21526, Egypt; summer.ashraf@alexu.edu.eg (S.A.A.); hesham25166@alexu.edu.eg (H.S.); 2 Program of Molecular Biotechnology, Faculty of Advanced Basic Sciences, Alamein International University, New Alamein City, Marsa Matrouh 5060310, Egypt; 3 Lung Microenvironmental Niche in Cancerogenesis, Institute for Lung Health (ILH), Justus Liebig University, 35390 Giessen, Germany

**Keywords:** PD-L1, KRAS, CRISPR, lung, cancer

## Abstract

Cancer cells can escape death and surveillance by the host immune system in various ways. Programmed cell death ligand 1 (PD-L1) is a transmembrane protein that is expressed by most cell types, including cancer cells, and can provide an inhibitory signal to its receptor PD-1, which is expressed on the surface of activated T cells, impairing the immune response. PD-L1/PD-1-mediated immune evasion is observed in several KRAS-mutated cancers. In the current study, we used the CRISPR/Cas9 system to knock down PD-L1 and KRAS in adenocarcinoma lung cells (A549 and H1975). Knockdown of PD-L1 was validated by qPCR and coculture with lymphocytes. The cells were functionally analyzed for cell cycle, migration and apoptosis. In addition, the effects of PD-L1 and KRAS downregulation on chemotherapy sensitivity and expression of inflammatory markers were investigated. Suppression of PD-L1 and KRAS led to a slowdown of the cell cycle in the G0/G1 phase and reduced migration, increased sensitivity to chemotherapy and triggered apoptosis of cancer cells. In addition, the conditioned medium of the modulated cells significantly affected the native cancer cells and reduced their viability and drug resistance. Our study suggests that dual silencing of PD-L1 and KRAS by CRISPR/Cas9 may be a promising therapeutic approach for the treatment of lung cancer.

## 1. Introduction

Tumors are composed of cancer cells that, together with other cell types, form a rich microenvironment that greatly contributes to tumor progression. Although tumors are able to evade surveillance by the immune system in many ways, the immune system has an essential function in limiting cancer [1]. Immune tolerance is fundamental to the development and spread of cancer and can lead to resistance to immunotherapies. For this reason, the discovery of therapeutic approaches to circumvent these immune resistance pathways in various malignancies is of great interest [2].

In recent years, promising results have been achieved in cancer immunotherapy using immune checkpoint blockade. Programmed cell death protein 1 (PD-1) and its ligand, the transmembrane protein PD-L1, are responsible for the interaction between T lymphocytes and cancer cells, leading to immune evasion and cancer tolerance. Blocking the PD-1/PD-L1 axis would reactivate the immune response and impede tumor progression.

PD-L1 is a small integral glycoprotein (33 kDa) that is present on cancer cells and has several aliases, such as CD274 and B7 homolog 1 (B7-H1). Normally expressed on macrophages, dendritic cells and some activated T cells, in the case of cancer it is expressed on the surface of tumor cells to evade the immune response [3,4]. Since PD-L1 is considered a pro-tumorigenic factor, therapeutic antibodies against PD-L1/PD-1 are attracting great attention because they impair tumor progression. Anti-PD-1 antibodies and anti-PD-L1 antibodies are examples of PD-1/PD-L1 checkpoint blockade immunotherapy strategies that have achieved a clinically meaningful anti-tumor effect [5,6]. Clinical trials in patients with solid tumors, such as non-small cell lung cancer and melanoma, showed a promising response to anti-PD-1/PD-L1 therapy [6]. Increased PD-L1 expression has been associated with metastasis, tumor progression and shorter survival rates [7,8].

However, immunotherapy with monoclonal antibodies is very expensive, especially as the production of antibodies is both difficult and time-consuming [9]. In addition, resistance to PD-1/PD-L1 immunotherapy and relatively low response rates in cancer are currently more common. It has been found that the efficacy of PD-1/PD-L1 blockade therapy is only ≤40% in several cancer types [7]. The reason for this limitation in antibody treatment is that PD-L1 has additional intrinsic regulatory (non-immunological checkpoint) functions that may play a critical role in promoting tumorigenesis and progression [8]. Genetic intervention using the CRISPR/Cas system therefore represents an alternative to the elimination of PD-1 and PD-L1. This system has been used both in vitro and in vivo, with PD-L1 being knocked out in mice [10]. In glioblastoma cells, CRISPR/Cas-mediated silencing of PD-L1 using two sgRNA sequences and a homology-directed repair (HDR) template inhibited proliferation and invasion [11]. Despite the promising results of anti-PD-L1 therapy, a considerable subgroup of patients does not respond to the therapy and develops resistance. Several studies have shown an increase in PD-L1 expression upon activation of the PI3K/AKT and RAS/MAPK signaling pathways, suggesting regulation of PD-L1 expression by extracellular signals such as hypoxia and cytokines that activate these pathways. In a study of triple negative breast cancer cells, blockade of the PD-1/PD-L1 axis led to inhibition of Akt and ERK phosphorylation and upregulation of p21 [12].

One frequent driver mutation in several cancers is known as the Kirsten rat sarcoma viral oncogene homolog (KRAS) [9]. The RAS gene family includes KRAS, a gene encoding a tiny membrane-bound GTPase that exists in one of two states: the active GTP-bound state, which significantly stimulates downstream signaling cascades that control essential cellular activities, or the inactive GDP-bound state. KRAS mutations cause constitutively active GTP-bound proteins that activate carcinogenic signaling pathways [13]. Direct suppression of KRAS by pharmacological agents has proven to be difficult. Nowadays, standard chemotherapy is used to treat KRAS-mutated tumors, although the success rate is often quite low [13,14]. A previous study has shown that in lung adenocarcinomas, p-ERK signaling can trigger PD-L1 expression in the presence of a KRAS mutation. In human KRAS-mutated tumors, blocking the PD-1/PD-L1 mechanism could represent an important therapeutic approach [15].

In this study, CRISPR/Cas9-mediated knockdown of PD-L1 and KRAS was investigated as a therapeutic approach for the treatment of lung cancer. The CRISPR/Cas9 system was used to disrupt PD-L1 and KRAS expression, and the effects of PD-L1 and/or KRAS knockdown on cell migration, apoptotic activity and drug resistance to the chemotherapies paclitaxel and L-asparaginase were investigated.

## 2. Results

### 2.1. PD-L1 KD Mediated by CRISPR/Cas9 Reduced Cell Viability and Increased Cell Sensitivity to Paclitaxel

**Figure 1:**
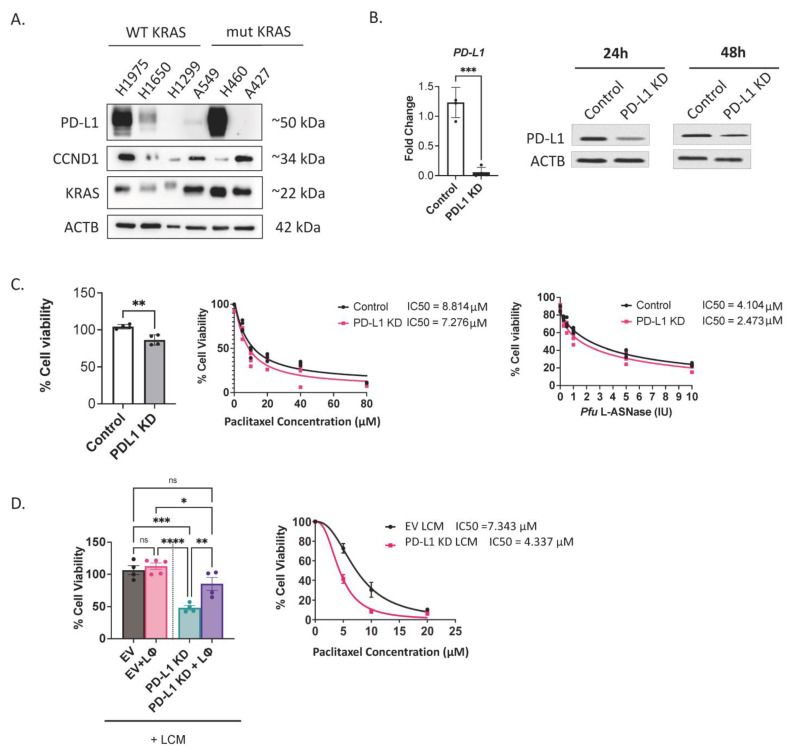
PD-L1 KD in A549 cells decreased cell viability and increased sensitivity to chemotherapy. (**A**) Screening of basal expression of PD-L1, KRAS and CCND1 by Western blot in KRAS wildtype cell lines and KRAS mutant non-small cell lung cancer cell lines using ACTB as a loading control. (**B**) PD-L1 expression analysis. Left panel: Expression analysis by quantitative real-time PCR of PD-L1 (*CD274*) in A549 cells after CRISPR/Cas9-mediated knockdown of PD-L1. Gene expression was normalized to *HPRT* as a housekeeping gene. Data represented as fold change to cells with control vector (*n* = 3). Right panel: Western blot showing PD-L1 expression 24 h and 48 h post-transfection using ACTB as loading control. (**C**) Cell viability tested by MTT assay after 24 h of plasmid transfections (*n* = 4) without treatment (left panel), after 24 h of treatment with paclitaxel (20 µM, 40 µM, 60 µM and 80 µM) (middle panel) and after treatment with L-ASNase *Pfu* (0.25 IU, 0.5 IU, 1 IU, 5 IU, 10 IU) (right panel). (**D**) Conditioned media of PD-L1 KD cells cocultured with lymphocytes decreased native A549 cells’ viability and increased their sensitivity to chemotherapy. Left panel: Cell viability of A549 cells tested by MTT assay using the lymphocyte-conditioned media (LCM) from A549 cells transfected with EV or PD-L1 KD cultured with and without lymphocytes (*n* = 4). Right panel: Cytotoxicity assay by MTT of native A549 while using LCM from control cells and cells with PD-L1 KD and treatment with 5, 10, 20 µM paclitaxel (*n* = 5). * *p* < 0.05, ** *p* < 0.01, *** *p* ≤ 0.001, **** *p* ≤ 0.0001.

As PD-L1 is an important communication marker on cancer cells, different lung cancer cell lines were screened for their basal expression of PD-L1. The KRAS mutational status was taken into consideration in the cell lines selected for screening. The KRAS protein is a GTPase that activates the MAPK pathway by binding to GTP. KRAS is known to be a key player in progression of many cancers including lung cancer, either owing to several missense mutations or due to its overexpression [16]. Even wildtype KRAS has a strong proliferative drive on cancer cells. It was of interest to screen the expression of PD-L1 in different lung adenocarcinoma cell lines with wildtype KRAS (H1975, H1650 and H1299) and cell lines harboring KRAS mutations (A549, H460 and A427). Among the WT KRAS cell lines, H1975 had the highest expression level of PD-L1, whereas H460 showed a similar high expression level among cell lines harboring KRAS mutation and H427 had almost no detectable expression of PD-L1 (Figure 1A). The A549 cell line carries mutant KRAS but had a traceable expression of PD-L1. Besides being the most studies model for lung adenocarcinomas, A549 cells were chosen for knocking down PD-L1 also because they had PD-L1 expression and a high level of CCND1 expression suggesting active proliferative drive.

Using the CRISPR/Cas9 system, PD-L1 was knocked down in A549 cells, which led to more than 90% reduction in PD-L1 expression on an RNA level (Figure 1B, right panel) and on a protein level (Figure 1B, left panel). As the transfection was transient, there was time-dependent decay in the knockdown efficiency over time post-transfection. PD-L1 knockdown decreased cell viability and sensitized A549 cells to both paclitaxel treatment and starvation by L-asparaginase (Figure 1C). L-asparaginase is an enzyme used in the treatment of leukemia because leukemic cells are dependent on L-asparagine for survival, but they lack asparagine synthetase. Despite presence of asparagine synthetase in A549 cells, starvation by L-asparaginase was found to increase cytotoxicity to different drugs.

PD-L1 is a ligand on cancer cell that interacts with PD-1 on T-lymphocytes, so it was of interest to evaluate this interaction after PD-L1 KD. For this purpose, lymphocytes were cocultured with naive A549 cells transfected with an empty vector as control and A549 cells transfected with PD-L1 KD plasmids. A lymphocyte-conditioned medium (LCM) of PD-L1 KD A549 cells reduced the viability of A549 cells (Figure 1D). However, direct coculture of cancer cells with lymphocytes ameliorated this effect, suggesting that the interaction is mainly mediated by the cytokines secreted into the milieu (Figure 1D). Furthermore, lymphocyte-conditioned medium collected after incubation with A549 cells with PD-L1 KD increased the sensitivity of native A549 cells to paclitaxel compared to the LCM of control cells (Figure 1D, right panel).

### 2.2. Expression Profile of Lung Cancer Cells upon PD-L1 and KRAS Knockdown

**Figure 2:**
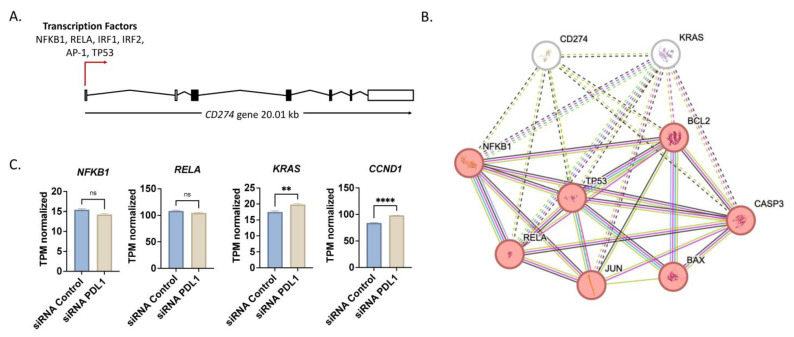
In silico analysis of the PD-L1 gene and correlation to expression upon PD-L1 knockdown in A549 cells reveal relation to apoptosis. (**A**) Schematic representation of the promoter of PD-L1 (*CD274*) gene (1000 bp upstream transcription start site) showing predicted transcription factors. (**B**) Protein–protein interaction network with DBSCAN clustering of transcription factors and apoptotic markers. Two clusters were identified by DBSCAN; the red nodes and the white nodes. Dotted lines represent interaction between different clusters whereas solid lines represent interaction within the same cluster. (**C**) Expression profile of selected genes upon silencing of PD-L1 by siRNA in erlotinib-resistant PC-9 (PC-9ER). Data obtained from GSE171650 by GEO2R are represented as transcripts per kilobase million (TPM). ** *p* < 0.01 and **** *p* < 0.0001.

PD-1/PD-L1 axis is regulated by several factors to orchestrate immune evasion mediated by lymphocytes. By screening the promoter region of *PD-L1* gene (*CD274*) using ALGGEN Research Software (https://alggen.lsi.upc.edu, accessed on 20 September 2023), transcription factor binding sites of inflammatory genes like *NFKB1*, *RELA* and *IRFs* were identified, in addition to binding sites for *TP53* and *AP-1* (Figure 2A). To visualize the interaction between these transcription factors and apoptosis in relation to CD274 and KRAS, a protein–protein interaction network (PPI) was generated using a STRING tool. Clustering by unsupervised density-based spatial clustering (DBSCAN) clustered CD274 with KRAS alone (white nodes) and other transcription factors, and the apoptotic markers BAX, BCL2 and CASP3 formed another cluster (red nodes, Figure 2B). Interestingly, KRAS was a key connection between CD274, inflammatory markers and apoptotic markers as well.

Owing to the activation of apoptosis by silencing of PD-L1, several studies have attempted genetic downregulation of PD-L1, such as the study by Ghosh et al. [17], where PD-L1 was knocked down using siRNA in erlotinib-resistant PC-9 (PC-9ER) non-small lung cancer cells [17,18]. Using their publicly available transcriptomic datasets from the GEO database (GSE171650), we investigated the expression of key inflammatory genes, namely *NFKB1* and *RELA*, as well as genes with proliferative drive as *KRAS* and *CCND1* (Figure 2C). Interestingly, in their study *KRAS* and *CCND1* were both upregulated upon silencing of PD-L1, whereas *NFKB1* and *RELA* were only slightly but insignificantly downregulated in their data.

**Figure 3:**
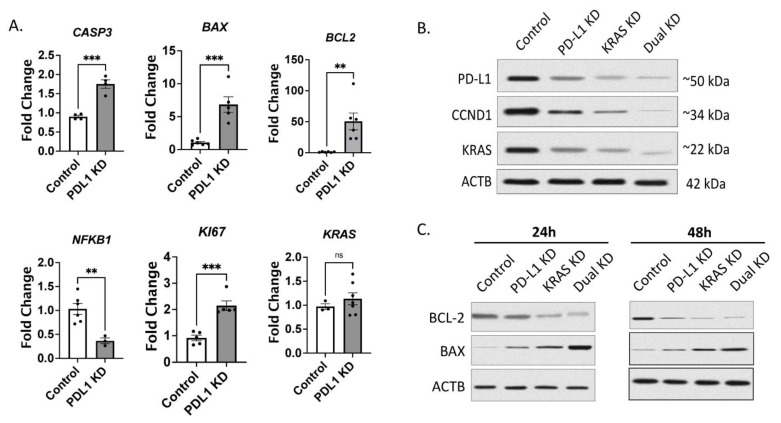
PD-L1 silencing in non-small lung cancer cells altered the expression of proliferation and apoptosis markers. (**A**) Expression analysis by qPCR in A549 cells after knocking down PD-L1 by CRISPR/Cas9 vector. Data represented as fold change to cells with control vector (*n* = 3–5). Statistical significance was tested by Student *t* test with ** *p* ≤ 0.01, *** *p* ≤ 0.001. (**B**) Expression profile by Western blot of A549 cells transfected with either PD-L1 CRISPR/Cas or KRAS CRISPR/Cas or both for 24 h showing proliferation markers and (**C**) apoptosis markers 24 h and 48 h post-transfection using ACTB as loading control.

Comparing their results with our system of CRISPR/Cas9-mediated knockdown of PD-L1 in A549 cells, qPCR data revealed that in concordance with their findings the inflammatory transcription factor *NFKB1* was downregulated whereas the apoptotic markers *CASP3*, *BAX* and *BCL2* and the proliferation marker *KI67* were upregulated (Figure 3A). The controversial increase of *KI67* expression was intriguing; however, previous studies have shown a controversial correlation between the proliferative marker KI67 and apoptosis. Lastly, KRAS was rather unchanged on an mRNA level.

As both PD-L1 and KRAS are known to have a proliferative anti-apoptotic drive, it was of interest to knock down both *KRAS* and *PD-L1* separately and combined in A549 cells. Indeed, protein expression analysis showed that both PD-L1 KD and KRAS KD elicited an antiproliferative switch that was potentiated upon simultaneous dual KD as seen in the lower expression of PD-L1, KRAS and CCND1 on a protein level (Figure 3B). In addition, pro-apoptotic marker BAX was upregulated in either single knockdown but stronger upon dual knockdown. Similarly, its counter-partner, the anti-apoptotic BCL-2, was downregulated to a greater extent upon dual knockdown (Figure 3C).

### 2.3. Dual Knockdown of PD-L1 and KRAS Induced Apoptosis and Caused Cell Cycle Arrest

**Figure 4:**
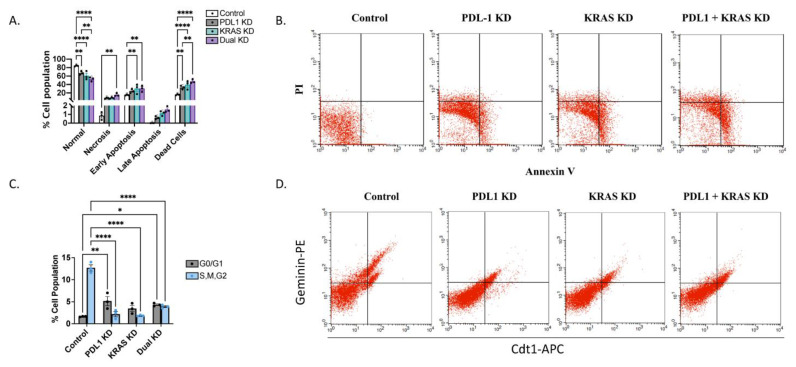
The single or double silencing of KRAS and PD-L1 increased apoptosis and arrested the cell cycle. (**A**) Apoptosis analysis by flow cytometry in A549 cells that were stained with annexin V-FITC and PI after 48 h of transfection (*n* = 3). (**B**) Dot plot showing representative graphs of cell distribution based on their extent of staining. Dots in the lower right quadrant represent cells in early apoptosis, and dots in the upper right quadrant represent cells in late apoptosis. (**C**) Cell cycle analysis by FUCCI system in A549 cells transfected with empty vector control or with PD-L1 knockdown cells (PD-L1 KD) or KRAS knockdown group (KRAS KD) or dual knockdown group in which both PD-L1 and KRAS genes were knocked down (PD-L1 + KRAS KD) (*n* = 3). Data represented as percentage cell populations in different phases of the cell cycle. (**D**) Dot plot of A549 cells showing fluorescence form mCherry-Cdt1 (APC) and citrine-geminin (PE). (Statistical significance was calculated by two-way ANOVA with * *p* ≤ 0.05, ** *p* ≤ 0.01, **** *p* ≤ 0.0001.)

Escaping cell death has been a well-established hallmark of cancer. Apoptosis analysis was performed to further elucidate the mechanism by which our target genes inhibit A549 cell viability. Apoptotic cells were measured by Annexin V/Propidium iodide double staining using flow cytometry after 48 h of plasmid transfection. As shown in Figure 4A, the proportion of dead cells in case of PD-L1 KD, KRAS KD and dual silencing of both genes was 32.836%, 39.356% and 47.266%, respectively, while control group had 15.776% dead cells. These results suggest that dual silencing of both PD-L1 and KRAS induced apoptosis more than silencing of each gene alone (Figure 4A,B).

To have a closer look on the effects of dual knockdown on the cell cycle, cells were transfected with FUCCI vector. The results revealed an increased percentage of the G0/G1 phase in PD-L1 KD cells (5.225%) and KRAS KD cells (3.485%) compared to control cells (1.690%) transfected with an empty vector as control (Figure 4C,D). Dual silencing of both PD-L1 and KRAS also induced the increase of cell population percentage in the G0/G1 phase (4.690%). We noticed that the percentage of cell population in G0/G1 phase was the lowest in the KRAS KD group. PD-L1 KD arrested the cells in G0/G1 phase to a greater extent than KRAS KD or the dual KD. This is probably due to the apoptotic effect of the knockdown.

### 2.4. PD-L1 Knockdown Impaired Migratory Capacity of A549 Cells

**Figure 5:**
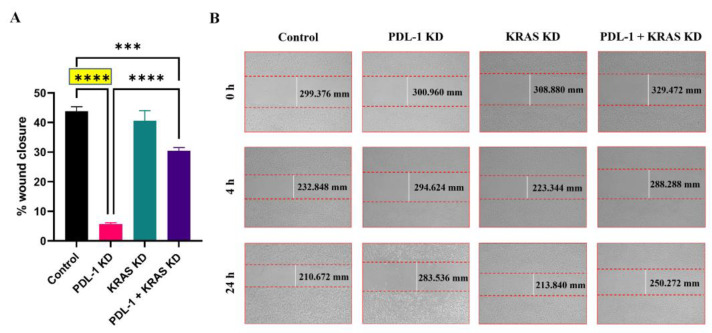
Silencing of KRAS abrogates the impaired migration upon PD-L1 knockdown. (**A**) Wound-healing migration assay in percentage of empty vector control-transfected A549 cells compared to PD-L1 knockdown group (PD-L1 KD) or KRAS knockdown group (KRAS KD) or dual knockdown group (PD-L1 + KRAS KD). Wound closure was quantified relative to zero time. (**B**) Representative images showing wound closure at zero time, 4 h and 24 h (10× magnification). Data are expressed as mean ± SEM (*n* = 3), and statistical significance was assessed by one-way ANOVA test with *** *p* ≤ 0.001, **** *p* ≤ 0.0001.

To investigate whether blocking of PD-L1 and KRAS would affect cell migration, a wound-healing assay was performed in 6-well plates. Interestingly, the genetic silencing of PD-L1 had the strongest inhibitory effect on the migration of A549 cells. As shown in Figure 5, PD-L1 KD, KRAS KD and their dual silencing inhibited cells migration by 38.19%, 3.317% and 13.38%, respectively. On the other hand, the effect of KRAS KD was modest, which even weakened the inhibitory effect upon dual silencing of PD-L1 and KRAS. These results indicate that the silencing of PD-L1 dramatically decreased cell migration, which may suggest that PD-L1 is a promoting factor for metastasis.

### 2.5. Dual Knockdown of PD-L1 and KRAS Improved Cytotoxicity of Paclitaxel Even in Cells with Wildtype KRAS

**Figure 6:**
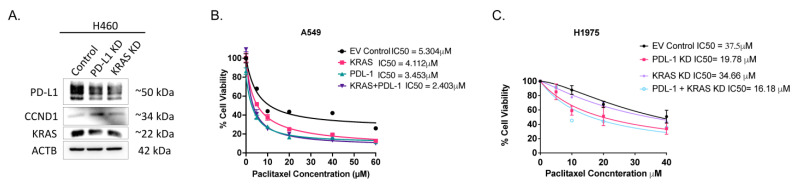
PD-L and KRAS dual knockdown in H460, A549 and H1975. (**A**) Expression profile by Western blot of H460 cells transfected with either PD-L1 CRISPR/Cas or KRAS CRISPR/Cas for 24 h using ACTB as loading control. (**B**) Cytotoxicity measured by MTT assay in adenocarcinoma cell lines A549 cells (*p* < 0.001) and (**C**) H1975 (*p* < 0.001) transfected with control vector, PD-L1 KD CRISPR/Cas9 vector, KRAS KD CRISPR/Cas9 vector or both. After transfection, cells were treated by paclitaxel for a further 24 h (*n* = 5).

As dual knockdown of both PD-L1 and KRAS had a noticeable effect on migration, apoptosis and cell cycle, it was of interest to verify this strategy on other cell lines considering their mutational burden. The initial screen for the expression of PD-L1 in different lung adenocarcinoma cell lines with wildtype KRAS (H1975, H1650 and H1299) and cell lines harboring KRAS mutations (A549, H460 and A427) showed the H460 and H1975 had the highest PD-L1 expression. The cell line H1975 harbored wildtype KRAS while H460 had mutant KRAS (Figure 1A). A549 cells are adenocarcinoma cells that harbor KRAS G12S mutation but wildtype EGFR, whereas H1975 harbors EGFR mutations L858R and T790M. It was of interest to compare the effect of PD-L1 and KRAS silencing in these adenocarcinoma cell lines. Both PD-L1 and KRAS expression were downregulated upon knockdown of either PD-L1 or KRAS in H460 cells. Interestingly, CCDN1 was slightly upregulated upon PD-L1 KD in H460 cells (Figure 6A). This is like the behavior of A549 cells, which were less proliferative and more apoptotic upon silencing of either PD-L1 or KRAS, besides being more sensitive to the chemotherapeutic agent paclitaxel (Figure 3B). Hence, it would be plausible to target both PD-L1 and KRAS in other cell lines with KRAS mutation.

When comparing A549 cells, which carry KRAS mutation, with H1975, which express wildtype KRAS, it was noticed that A549 cells were generally more sensitive to paclitaxel than H1975 cells. A549 cells had an IC50 of 5.3 µM whereas H1975 cells had an IC50 of 37.9 µM without any silencing (Figure 6B and Figure 6C, respectively). Interestingly, although KRAS KD had a minute effect on H1975 (IC50 of 36.22 µM), dual silencing of both PD-L1 and KRAS was more effective (IC50 of 16.18 µM) than either gene alone. This implies the importance of therapeutically targeting both genes even in absence of KRAS mutation.

## 3. Discussion

Immune checkpoint blockade remains a promising therapy despite the unpredictable possibility of resistance in almost half of lung cancer patients [19]. Several anti-PD-1/PD-L1 antibodies such as pembrolizumab, atezolizumab and nivolumab have been approved by the FDA for the treatment of non-small cell lung cancer and other cancers such as melanoma, kidney and breast cancer. However, their cost is a limitation, and their therapeutic efficacy has been associated with inflammatory side effects, making specific gene-editing tools such as CRISPR/Cas systems more attractive [20].

In this study, CRISPR/Cas-mediated knockdown of PD-L1 in A549 cells made them more susceptible to recognition by T lymphocytes and subsequent death (Figure 1). Lymphocytes cultured with A549 cells with low PD-L1 expression secreted a different set of cytokines that tended to reduce cancer cell cytotoxicity even in the absence of T lymphocytes. As the transfection efficiency was 60–80%, the cytotoxic effect of lymphocytes was ameliorated.

Although immune checkpoint blockade is an attractive therapeutic approach, several mechanisms have been hypothesized as causes for the unsatisfactory response, including the genetic variation landscape in key genes such as STK11/LKB1 and TP53, particularly in KRAS-mutated lung cancer, which accounts for 20–25% of lung cancers [19]. In addition, the non-immune effects of PD-1 and PD-L1 promote resistance to chemotherapy and drive the cells to metastasize. In kidney cancer, researchers suspect that PD-L1 promotes tumor progression by inducing the stem cell phenotype of cancer cells and thus the epithelial mesenchymal transition (EMT) [4].

In A549 cells, PD-L1 KD alone also had the strongest inhibitory effect on cell migration (Figure 5). Surprisingly, the effect of KRAS KD was minimal and attenuated the effect of PD-L1 on dual knockdown. It would be of great interest to transfect cells not only transiently but stably to investigate the long-term effects of KD in lung cancer patients with KRAS mutations. KRAS is known to be a strong driver of primary tumor growth, and the mutations are tumor-specific. In both lung cancer and colorectal cancer, the KRAS mutation pattern is quite heterogeneous between the primary tumor and the corresponding metastases. This heterogeneity requires a more detailed analysis and screening of patient tumors [21].

Interestingly, in our study, KRAS KD mainly affected apoptosis and the cell cycle, while PD-L1-KD had the strongest inhibitory effect on migration. Since cancer is a multifactorial disease with a plethora of altered signaling, several treatment modalities attempt combination therapy. In renal carcinoma, the oral tyrosine kinase inhibitor (TKI) anlotinib, which targets multiple signaling pathways, namely vascular endothelial growth factor receptor (VEGFR) and fibroblast growth factor receptor (FGFR), has been used in conjunction with anti-PD-L1 antibodies [22]. Another oral TKI, axitinib, was combined with anti-PD-L1 in hepatocellular carcinoma and achieved encouraging results. Sorafinib was also approved by the FDA together with immune checkpoint inhibitors [23].

Taken together, targeted inhibition of PD-L1 and KRAS seems very plausible, firstly because immune checkpoint blockade is often more successful in cancers with high tumor burden such as KRAS-mutated tumors. Secondly, these tumors often show immune evasion, as KRAS signaling upregulates immunosuppressive factors such as IL-10 and TGF-ß and activates CD47 [21,24]. Consistent with this logic, our results also showed that silencing KRAS significantly decreased proliferation, increased apoptosis and sensitized cells to chemotherapy. Dual targeting of KRAS and PD-L1 could be beneficial in treating tumors with either wildtype or mutant KRAS. Our results in A549 and H460 were consistent and even H1975 was more responsive to chemotherapy upon dual silencing of PD-L1 and KRAS. Targeting oncogenic RAS signaling and PD-L1 expression in adenocarcinomas could overcome immunoresistance [25]. Interfering with both signaling pathways has potential for the treatment of lung cancer heterogeneity.

## 4. Materials and Methods

### 4.1. Cell Culture

The A549 and H1975 cell lines were obtained from the cell culture facility at Center of Excellence for Research in Regenerative Medicine and Applications (CERRMA), Faculty of Medicine, Alexandria University and all cell culture experiments were conducted there. This study was conducted according to the research codes of ethics followed at the Faculty of Medicine, Alexandria University. Lung cancer cell lines H1650, H1299, A427 and H460 were obtained from ATCC and experiments were performed at the cell culture facility at Lung Microenvironmental Niche in Cancerogenesis, Institute for Lung Health (ILH), Justus Liebig University, Giessen, Germany.

All cells were cultured in DMEM (4.5 g/L glucose) media supplemented with 10% fetal bovine serum (FBS) and 1% penicillin/streptomycin solution and kept in a humidified incubator with 5% carbon dioxide (CO_2_) at 37 °C. Cells were passaged every 2–3 days until they reached about 80–90% confluency. Cells were counted using hemocytometer in presence of 0.4% Trypan blue dye to detect dead cells. Unless otherwise specified, cells were seeded in 96-well plates at a density of 7000 cells/well and in 6-well plates at a density of 3 × 10^5^ cells/well [26].

### 4.2. Bacterial Transformation Plasmid Purification

#### 4.2.1. Preparation of *E. coli* Competent Cells

*E. coli* BL21(DE3)pLysS bacterial glycerol stock was activated overnight in LB broth at 37 °C with shaking (150 rpm). The culture was streaked on an LB plate and incubated overnight to isolate single colonies. A single colony was grown in 1 mL LB broth and used to inoculate 100 mL LB broth at an inoculum size of 1%. The culture was grown with shaking at 37 °C until the optical density at 600 nm reached an absorbance of 0.2–0.4, usually after 2–3 h. The growth was stopped by placing the culture on ice for 30 min. Cells were collected by centrifugation at 2000× *g*, at 4 °C for 20 min. The supernatant was decanted, the cells were suspended in 10 mL pre-cooled 0.1 M CaCl_2_ and kept on ice for 30 min. Cells were centrifuged again using the same conditions and the cell pellet was resuspended in 1–2 mL pre-cooled 0.1 M CaCl_2_. The competent cells were kept at 4 °C until use and for maximum 48 h [27].

#### 4.2.2. Transformation in *E. coli* Competent Cell

Three mammalian expression plasmids were amplified in *E. coli* to be used for the assessment of the gene modulation impact on the cultured cells; two pLentiCRISPR v2 vectors were used to deliver the CRISPR/Cas-9 system together with an empty pLentiCRISPR v2 as empty vector (EV).

The plasmid of interest (1 µL) was added to 50 µL competent cells, and cells were kept on ice for 30–40 min then heated at 42 °C for 40 s in a water bath. After heat shock, cells were kept on ice for 5 min and then 950 µL LB broth was added to the cells and incubated at 37 °C for 2–4 h with shaking. Cells (100–200 µL) were spread on an LB plate with ampicillin (100 µg/mL) and incubated overnight at 37 °C.

#### 4.2.3. Plasmid Preparation

After incubation, single colonies were selected to grow separately in LB broth overnight. The culture from several colonies was centrifuged to collect cell pellets. Cell pellets were resuspended in cell resuspension buffer, then 350 µL cell lysis buffer (0.2 N NaOH, % SDS) was added. After complete cell lysis, 350 µL neutralization buffer (1.32 M potassium acetate pH 4.8) was added, mixed well by vortexing, then put on ice for 10 min. To remove cell debris, the mixture was centrifuged at 7000× *g* for 10 min and the supernatant was transferred to another Eppendorf tube. Equal volume of isopropanol was added to the supernatant to precipitate plasmid DNA. After centrifugation at 7000× *g* for 10 min, the supernatant was discarded, and the DNA pellet was washed twice with 500 µL ethanol and left to air-dry. The plasmid was resuspended in 30–50 µL nuclease-free water, and its concentration was measured by Nanodrop (DeNovix, Wilmington, DE, USA).

### 4.3. Silencing of PD-L1 and KRAS

#### 4.3.1. CRISPR-Cas9 System

The CRISPR-Cas9 system was applied to silence PD-L1 and KRAS genes. We transfected the pLentiCRISPR v2 vector (GenScript, Cat#S58112), which encodes the sgRNA, into cells. The sgRNA sequences for PD-L1 and KRAS were TACCGCTGCATGATCAGCTA and TCTCGACACAGCAGGTCAAG, respectively. qPCR was used to validate gene knockdown.

#### 4.3.2. Plasmid Transfection

Cells were seeded in 96- and 6-well plates at density of 7 × 10^3^ cells/well and 3 × 10^5^ cells/well, respectively, and cultured overnight. When they reached 70–80% confluency, the media was subsequently removed. Cells were transfected with empty vector pLentiCRISPR v2 vector or pLentiCRISPR v2 vector with PD-L1 knockdown (PD-L1 KD), or pLentiCRISPR v2 vector for KRAS knockdown (KRAS KD), respectively, using Lipofectamine^®^ 2000 Transfection Reagent (Thermo Fisher Scientific, Dreieich, Germany) according to the manufacturer’s protocol. Briefly, DNA and Lipofectamine were prepared at a ratio of 1:3 (amount of plasmid DNA in µg:volume of transfection reagent in µL) in serum-free medium and added to the cells. After 24 h, the medium was replaced, and the transfection efficiency was assessed under the microscope according to the GFP expression. It was in the range 70–90% according to the cell line.

### 4.4. Analysis of Gene Expression by Quantitative Real-Time Polymerase Chain Reaction (qRT-PCR)

For RNA collection, cells were seeded in a 6-well plate and total RNA was isolated from cells using Trizol reagent (Qiagen, Hilden, Germany) following the manufacturer’s protocol. The purity and the concentration of RNA samples were determined using Nanodrop (DeNovix, Wilmington, DE, USA) and integrity of RNA was checked by agarose gel electrophoresis. An amount of 2 µg RNA was transcribed to cDNA using the High-Capacity cDNA Reverse Transcription Kit (Applied Biosystems, Darmstadt, Germany) according to the manufacturer’s instructions using random primers. The synthesized cDNA was diluted 1:1 with nuclease-free water before proceeding with qPCR and stored at −20 °C until use.

A real-time qPCR reaction mixture was prepared using Maxima SYBR Green (Thermo Fisher Scientific, Dreieich, Germany) according to the manufacturer’s recommendations using following primers: *PD-L1* (*CD274*) FW: GGACAAGCAGTGACCATCAAG, *PD-L1* (*CD274*) RV: CCCAGAATTACCAAGTGAGTCCT; *KRAS* FW: CAGTAGACACAAAACAGGCTCAG, *KRAS* RV: TGTCGGATCTCCCTCACCAATG; *NFKB1* FW: GCCACCCGGCTTCAGAATGG, *NFKB1* RV: GGCCATCTGCTGTTGGCAGT; KI6 FW: GAGGTGTGCAGAAAATCCAAA, *KI67* RV: CTGTCCCTATGACTTCTGGTTGT; *CASP3* FW: TTTTTCAGAGGGGATCGTTG, *CASP3* RV: CGGCCTCCACTGGTATTTTA; *CASP8* FW: CCTGGGTGCGTCCACTTT, *CASP8* RV: CAAGGTTCAAGTGACCAACTCAAG; *BAX* FW: TTCATCCAGGATCGAGCAG, *BAX* RV: TGAGACACTCGCTCAGCTTC; *BCL2* FW: CACCTGTGGTCCACCTGAC, *BCL2* RV: ACGCTCTCCACACACATGAC; and *HPRT* FW: TGACACTGGCAAAACAAT, *HPRT* RV: GGTCCTTTTCACCAGCAA, which was used as housekeeping gene. The assay was run at 57 °C on an Applied Biosystems StepOne^TM^ Instrument. The expression of genes of interest was normalized to the expression level of reference gene *HPRT* and fold change of the target gene was calculated relative to the empty vector control sample.

### 4.5. Cytotoxicity Assay by MTT

Cells were seeded in 96-well plates at density of 7 × 10^3^ cells/well and cultured overnight to attach. When they reached 70–80% confluency, the media was removed and replaced with media containing paclitaxel or L-ASNase Pfu at different concentrations. After incubation for 24 h at 37 °C, 10 µL of MTT (3-(4,5-dimethylthiazol-2-yl)-2,5-diphenyltetrazolium bromide, 5 mg/mL) was added to each well and cells were incubated for 4 h. The MTT solution was then removed, and the formed formazan crystals were solubilized in 100 μL of DMSO. After shaking the plates for 20 min at room temperature, the optical density (OD) was measured at 570 nm utilizing a microplate reader (Tecan, San Jose, CA, USA). The percentage cell viability was calculated relative to the control native cells and the half-maximal inhibitory concentrations (IC50) were deduced from the respective sigmoidal concentration-response curve plotted using the following equation [28].

$$\%\;cell\;viability=\frac{absorbance\;of\;modulated\;cell}{absorbance\;of\;control\;cells\;(average)}\times 100$$

### 4.6. Expression Analysis by Western Blotting

Cells were transfected with respective plasmids for 48 h before proteins were collected in RIPA buffer supplemented by protease and phosphatase inhibitors. After protein quantification by Lowry assay, 20 µg protein samples were loaded on SDS-PAGE gels for Western blotting using the following antibodies: PD-L1 (#MA5-27896, Invitrogen, Waltham, MA, USA), KRAS (#ab55391, Abcam, Cambridge, UK), CCDN1 (#2978S, Cell Signalling, Danvers, MA, USA) and ACTB (#ab6276, Abcam) as loading controls.

### 4.7. Migration Assay by Wound-Healing Scratch Assay

For migration assay, cells were seeded in 6-well plates at a density of 3 × 10^5^ cells/well and incubated for 24 h. After plasmid transfection, a 200 µL pipette tip was used to make a scratch in the middle of each well. A phase-contrast microscope was used to record the wound areas at 0, 4 and 24 h. Microscopic images were taken at 10× magnification. Cells were cultured overnight at 37 °C for 24 h and again microscopic images were taken to assess the wound closure. The migration capability was evaluated by measuring the migration distance and percentage wound closure. Wound closure was quantified as the percentage by which the initial scratch width has decreased for each given time point [29]. The experiment was carried out in triplicates and data were represented as mean ± SEM. Images were analyzed using ImageJ software, version 1.51j8, and the percentage wound closure was calculated as follows:

$$\%\;wound\;closure=\frac{wound\;size\;at\;0\;h-wound\;size\;at\;24\;h}{wound\;size\;at\;0\;h}\times 100$$

### 4.8. Apoptosis Detection Using Flow Cytometer

Apoptosis testing was carried out using the Annexin V-Fluorescein Isothiocyanate (FITC) Apoptosis Detection Kit. The control group include cells transfected with empty vector in addition to unstained cells to measure background fluorescence and to determine the gating for the analysis of the upcoming samples. After 48 h of transfection, cells were stained with PI alone, cells stained with annexin VFITC alone, cells stained with both PI and annexin V/FITC [30]. Cells were seeded at a density of 3 × 10^5^ in 6-well plates and incubated overnight at 37 °C and 5% CO_2_. The supernatants were decanted, and the cell pellets were washed twice with PBS and once with 1× binding buffer and resuspended in 1 mL 1× binding buffer. One hundred microliters was then transferred to a clean tube and 5 µL of Annexin V-FITC and 5 µL of PI were added and the tubes were incubated at room temperature for 15 min in the dark [29]. Finally, 400 µL of 1× binding buffer was added. Apoptosis was measured by using BD FACS Calibur flow cytometer and cell sorter and Cell Quest™ software version 5.1 according to the instructions provided by the Annexin V-FITC kit (Becton-Dickinson, Franklin Lakes, NJ, USA).

### 4.9. Cell Cycle Analysis Using FUCCI Assay

A549 cells were transfected with both CRISPR/Cas9 plasmid with either sgRNA for *PD-L1* or for *KRAS* or both, together with ES-FUCCI plasmid, which was a gift from Pierre Neveu (Addgene plasmid #62451; http://n2t.net/addgene:62451, accessed on 30 November 2020; RRID: Addgene_62451). After 48 h of transfection, cells were fixed and measured by flow cytometry to quantify for mCherry-Cdt1 and citrine-geminin [31].

### 4.10. Coculture of A549 with Lymphocytes

Lymphocytes were isolated by Ficoll centrifugation gradient from a human blood sample obtained from a healthy donor. They were collected separately from monocytes and used in their native state. Briefly, following isolation, lymphocytes were cultured in RPMI media and incubated overnight. Subsequently, lymphocytes were cultured with A549 cells having PD-L1 knocked down, or control A549 cells transfected with empty vector. After 24 h, the lymphocyte-conditioned medium (LCM) was harvested and centrifuged to remove lymphocytes. Supernatant was then added to native A549 cells before treatment with paclitaxel (7 µM) in a 96-well plate [32]. For direct coculture, lymphocytes were directly added to A549 cells after their transfection with the respective plasmids.

### 4.11. Statistical Analysis

Statistical analyses were performed with GraphPad Prism 9 Software (GraphPad Software, Inc., La Jolla, CA, USA). Student’s *t* test (two-tailed) was used to compare two groups. When more than two groups were compared, differences among the groups were determined by one-way ANOVA. Data are expressed mean ± SEM; statistical significance was set at *p* ≤ 0.05. Significance level is noted as follows: * *p* ≤ 0.05, ** *p* ≤ 0.01, *** *p* ≤ 0.001, **** *p* ≤ 0.0001 unless otherwise specified.

## 5. Conclusions

Genetic manipulation with CRISPR/Cas9 offers a “natural” way of gene editing, especially when it comes to PD-L1 on cancer cells or PD-1 on immune cells, as it mimics bacterial defense mechanisms against phages. However, targeting a gene presents the cell with the challenge of developing mechanisms to overcome these changes. PD-L1-KD via the CRISPR/Cas9 system led to upregulation of KRAS, so dual knockdown of PD-L1/KRAS is a logical therapeutic approach with promising results as it overrides potential immune evasion with current KRAS therapies.

## Data Availability

The data presented in this study are available on request from the corresponding author. Publicly available datasets were analyzed in this study. (https://www.ncbi.nlm.nih.gov/geo/query/acc.cgi?acc=GSE171650, accessed on 7 June 2024).
